# Text Mining Strategy Identifies Gene Networks Under Control of miR‐21 in Breast Cancer Development

**DOI:** 10.1002/cam4.70986

**Published:** 2025-06-26

**Authors:** Hong Ye, Yuyu Wu, Richard Tran, Jie Wang

**Affiliations:** ^1^ Department of Neurology Xiangshan Hospital of TCM Medical and Health Group Ningbo City Zhejiang Province China; ^2^ Department of Acupuncture Xiangshan Hospital of TCM Medical and Health Group Ningbo City Zhejiang Province China; ^3^ Masters Program in Computer Science University of Chicago Chicago Illinois USA; ^4^ Applied Data Science Program Syracuse University Syracuse New York USA

**Keywords:** apoptosis, breast cancer, miR‐21, miRNA, natural language processing, text mining

## Abstract

**Background:**

MicroRNAs (miRNAs) are small regulatory molecules that play a critical role in various biological processes by regulating gene expression. They have emerged as crucial players in cancer development, including breast cancer. However, individual research studies may be subject to specific biases.

**Methods:**

To gain a more comprehensive understanding of miRNA involvement in breast cancer, we employed a large‐scale analysis of miRNA studies retrieved from PubMed. Our approach involved tokenizing abstracts to identify key biomedical entities (e.g., miRNA, gene, disease) and constructing miRNA‐cancer co‐occurrence networks using bioinformatic analysis.

**Results:**

This analysis revealed miR‐21 as the most frequently studied miRNA in breast cancer research, with a significant difference compared to other miRNAs. Network analysis identified SMAD3, PIK3R1, STAT3, and TP53 as key regulators potentially affecting pathways like TGF‐β signaling and p53 signaling. Additionally, our analysis suggests that genes associated with miR‐21 are often downregulated in tumors and exhibit a positive correlation with T cell infiltration, particularly CD8+ T cells, potentially indicating a favorable prognosis.

**Conclusion:**

Our findings highlight miR‐21 as a central regulatory hub and potential biomarker in breast cancer. While informative, the results are derived from literature‐based data and may be influenced by text‐mining limitations, underscoring the need for experimental validation.

**Abbreviations**
ABCA1: ATP‐binding cassette sub‐family A member 1
ABCB1: ATP‐binding cassette sub‐family B member 1
AKT: protein kinase B
ANKRD46: Ankyrin repeat domain‐containing protein 46
AR: hormone androgen receptor
BAX: BCL2 associated X
BCL2: B‐cell lymphoma 2
BRCA1: breast cancer type 1 susceptibility protein
BRCA2: breast cancer type 2 susceptibility protein
CA15‐3: cancer antigen 15–3
CASP3: Caspase 3
CCNB1: Cyclin B1
CDC25A: cell division cycle 25A
CDK1: Cyclin‐dependent kinase 1
CDK2: Cyclin‐Dependent Kinase 2
CDK6: Cyclin‐dependent kinase 6
CEA: Carcinoembryonic antigen
CSF1: Colony stimulating factor 1
DAVID: The Database for Annotation, Visualization, and Integrated Discovery
DEGs: differentially expressed genes
EGFR2: epidermal growth factor receptor 2
EMT: epithelial‐mesenchymal transition
EPCAM: epithelial cell adhesion molecule
ER: estrogen receptor
ER: hormone estrogen receptor
EREG: epiregulin
FASLG: Fas ligand
FC: fold change
GSCA: gene set cancer analysis
HER2: human epidermal growth factor receptor 2
KDR: Kinase insert domain receptor
MAP K1: Mitogen‐activated protein kinase 1
miRNAs: microRNAs
MMP1: Matrix metalloproteinase 1
mRNA: messenger RNA
mTOR: mammalian target of rapamycin
NER: named entity recognition
NFKB1: Nuclear Factor Kappa B Subunit 1
NLP: natural language processing
PARP1: poly [ADP‐ribose] polymerase 1
PDCD4: programmed cell death protein 4
PIK3CA: phosphatidylinositol‐4,5‐bisphosphate 3‐kinase catalytic subunit alpha
PIK3R1: phosphoinositide‐3‐kinase regulatory subunit 1
PPI: protein–protein interaction
PR: progesterone receptor
PTEN: phosphatase and tensin homolog
RAS: rat sarcoma virus GTPase
RRM2: ribonucleoside‐diphosphate reductase subunit M2
RTK: Receptor Tyrosine Kinase
SMAD3: SMAD family member 3
SPRY1: Sprouty homolog 1
SPTBN1: Spectrin beta chain, non‐erythrocytic 1
STAT3: Signal transducer and activator of transcription 3
TCGA: The Cancer Genome Atlas Program
TGF‐beta: transforming growth factor beta
TIMER: Tumor Immune Estimation Resource
TIMP3: tissue inhibitor of metalloproteinases 3
TP53: tumor protein p53
TSC: tuberous sclerosis complex
VEGF: vascular endothelial growth factor
VIM: Vimentin
ZEB1: Zinc finger E‐box‐binding homeobox 1
ZNF367: Zinc finger protein 367

## 1 Introduction

The field of genomics has been revolutionized in recent years by powerful computational tools and the ever‐increasing volume of biomedical research [1, 2, 3]. This dynamic landscape has fostered the development of innovative approaches to unravel the molecular underpinnings of diseases [2, 4, 5]. Among these, microRNAs (miRNAs) have emerged as crucial players in post‐transcriptional gene regulation [6]. These small molecules, typically around 22 nucleotides long, bind to specific sequences within target messenger RNA (mRNA) molecules, influencing mRNA stability or translation [7]. Consequently, miRNAs play vital roles in development, health, and disease [8, 9]. Furthermore, miR‐21 is one of the most abundant and well‐studied miRNAs [10]. It is expressed in nearly all cells, where it plays critical regulatory roles in various diseases [11]. These include contributions to immune cell development [12, 13], autoimmune diseases [14, 15], and multiple cancers [16, 17, 18, 19, 20]. Additionally, miR‐21 has been identified as a potential biomarker for diseases like cancer and heart disease in studies analyzing miRNAs in bodily fluids [11].

Breast cancer remains a significant public health challenge. It is the most common malignancy diagnosed in women, accounting for 18% of all cancers and the second leading cause of cancer‐related deaths among women [21, 22]. While early detection through screening programs has improved survival rates, breast cancer research is a continuously evolving field due to the complex interplay of genetic, hormonal, and environmental factors [23]. Understanding the molecular mechanisms underlying breast cancer is crucial for developing targeted therapies and improving patient outcomes.

Several miRNAs, including miR‐21 [24, 25, 26], miR‐182 [27, 28, 29, 30], miR‐155 [31, 32, 33], miR‐3117 [34], miR‐143 [35], miR‐99a [35], miR‐140 [36], miR‐149 [37] have been demonstrated to play roles in breast cancer development and progression. In addition, bioengineered miRNAs have emerged as promising tools for targeted cancer therapy [38]. In this study, we propose to utilize text mining to systematically identify, compile, and analyze existing research on miR‐21 in breast cancer. By employing this innovative method, our objectives are (1) to generate a comprehensive understanding of the current knowledge landscape by analyzing a large corpus of studies, potentially uncovering hidden connections and insights, and (2) to bridge the gap between diverse studies, facilitating a more effective understanding of miR‐21's impact on breast cancer development and progression. While miR‐21 is the focal point of detailed analysis, our methodology and data collection included a comprehensive set of miRNAs, which lays the groundwork for future large‐scale investigations.

## 2 Materials and Methods

### 2.1 PubMed Corpus

We retrieved relevant literature for studies investigating miR‐21 in the context of cancer using PubMed. The search covered the period from January 1, 2010, to August 15, 2023. We employed the following search criteria: [[‘(miRNA‐21[Title] or miR‐21[Title]) AND (cancer [Title] or tumor or carcinoma or neoplasm or oncology or malignancy)’]]. Following the established approach from our previous studies [39, 40], we extracted the following information from each identified publication: “PubMed ID (PMID)”, “Publication Date”, “Publication Type”, “First Author”, “Journal Name”, “Literature Title”, and “Literature Abstract”. We specifically focused on independent research articles categorized as “Journal Article” within the publication type. Other types of publications, including comparative studies, review articles, meta‐analyses, comments, editorials, evaluation studies, letters, news items, published errata, retractions, and systematic reviews, were excluded from the analysis.

### 2.2 Biomedical Term Tagging From Literature Studies

Literature titles and abstracts are widely available and contain valuable information for biological research. Therefore, we focused on mining text from both the title and abstract for the current study. To tag and categorize biomedical entities within the literature, PubTator [41] was employed. This tool tags various entities in PubMed titles and abstracts, including genes, diseases, species, chemicals, cell lines, and mutations.

### 2.3 Gene ID Standardization

To ensure consistency in gene identification across different studies, all tagged genes were converted to their corresponding ENSEMBL Gene IDs using the Database for Annotation, Visualization, and Integrated Discovery (DAVID) Functional Annotation Tool (https://davidbioinformatics.nih.gov/) [42].

### 2.4 Data Export and Analysis

The results generated by the PubTator tool are exported in PubTator format, which can be accessed and processed using Python for further analysis and exploration. To avoid introducing bias, we only counted each tagged entity once within a given literature title/abstract. In the following analysis, only studies involving human subjects were included. Species tagged as “homo sapiens”, “human”, “Child”, “children”, “humans”, “men”, “participant”, “participants”, “patient”, “patients”, “people”, “persons”, “woman”, and “women” were categorized as human models, while the broader term, such as “mammalian,” was excluded to ensure accurate representation of the species used in our study.

### 2.5 Functional Analysis of Hub Genes

To explore the comprehensive functional landscape of the identified hub genes, we employed two complementary approaches: protein–protein interaction (PPI) network analysis and gene set enrichment analysis (MSigDB Hallmark analysis). We applied the hypergeometric test to identify significantly enriched pathways. The PPI networks were generated using STRING [43] (version 12.0, https://string‐db.org/), and clustering calculation and visualization were generated using Gephi software (version 0.10, https://gephi.org/) [44].

### 2.6 Analysis of Differentially Expressed Genes (DEGs)

Differentially expressed genes (DEGs) between breast cancer and normal tissue samples were obtained from The Cancer Genome Atlas Program (TCGA) dataset [45] (https://www.cancer.gov/ccg/research/genome‐sequencing/tcga), based on a significant threshold of *p* < 0.05. The top 250 upregulated and 250 downregulated genes in breast cancer were selected for further analysis. Additionally, we analyzed the correlation between gene expression and tumor purity, as well as T cell infiltration, using the Tumor Immune Estimation Resource (TIMER) tool [46] (https://cistrome.shinyapps.io/timer/). Finally, to gain deeper insights into the functional roles of the genes in breast cancer, we performed gene set cancer analysis (GSCA) using the GSCA tool [47, 48] (http://bioinfo.life.hust.edu.cn/GSCA/).

### 2.7 miRNA Motif Analysis

miRNA motif analysis was performed using the NetworkAnalyst platform (https://www.networkanalyst.ca/). Starting with a gene list, we utilized the “Gene Regulatory Networks” module and accessed “Gene‐miRNA Interactions.” This module leverages miRTarBase v9.0, a comprehensive database of experimentally validated miRNA‐target interactions. The analysis identifies enriched miRNA motifs within the 3′ untranslated regions (UTRs) of target genes, providing insights into potential regulatory mechanisms.

### 2.8 Statistics Analysis

GraphPad Prism 9.0 was used for statistical analysis (unpaired, two‐tailed, *t*‐test with 95% confidence interval). A *p*‐value of < 0.05 was considered significant.

## 3 Results

### 3.1 Study Design and Workflow

**FIGURE 1:**
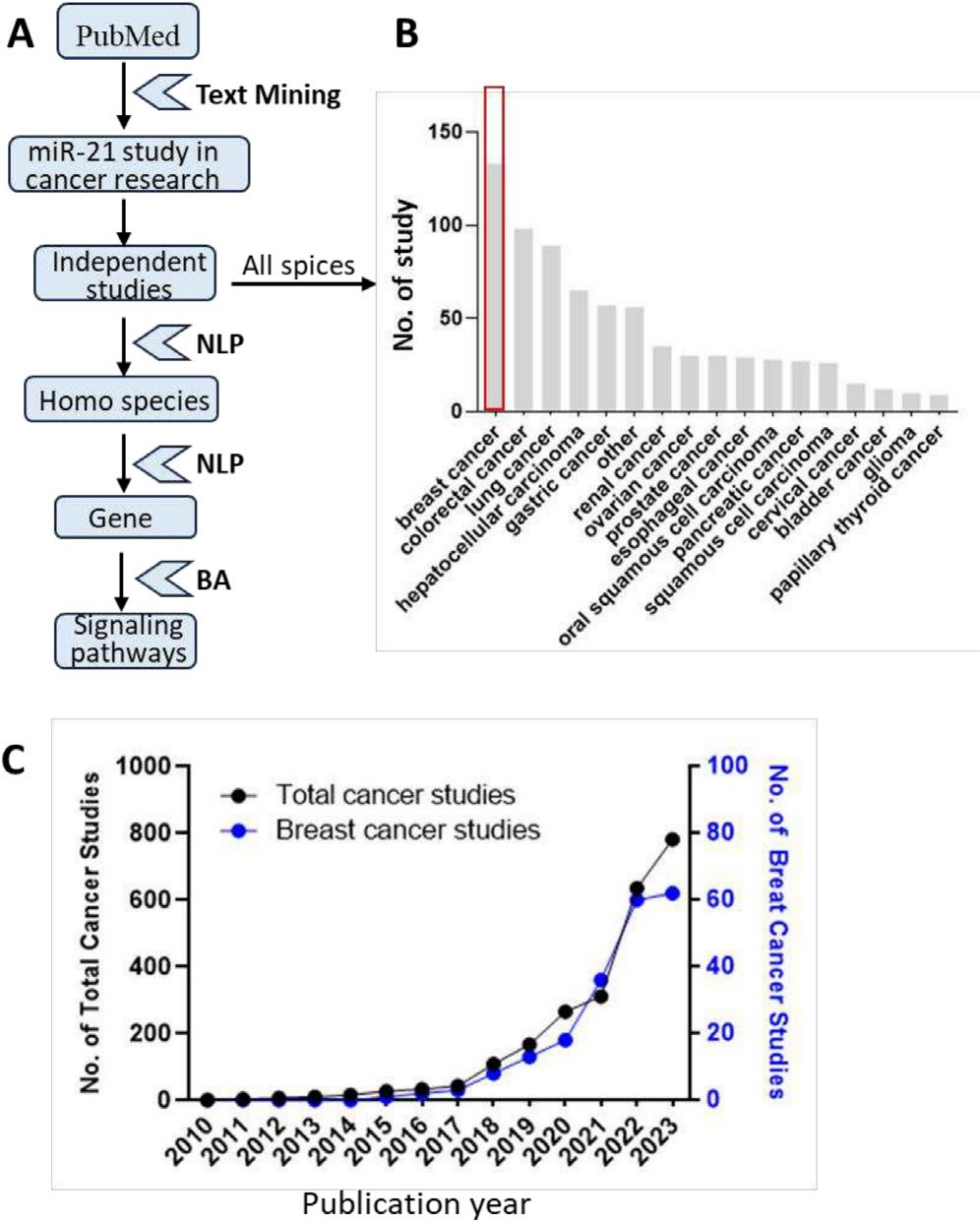
Study strategy. (A) Study design and workflow. (B) The number of miR‐21 studies on different cancer research. (C) Comparison of frequency distribution of miR‐21 in total cancer studies (black) and in breast cancer studies (blue).

As shown in Figure 1A, this study presents a framework for identifying miRNA biomarkers by exploring the involvement of miRNA‐gene pairs in cancer research through biomedical text mining and natural language processing (NLP). First, cancer‐related miRNA studies were extracted from PubMed using specific terms such as ‘cancer’, ‘tumor’, ‘carcinoma’, ‘neoplasm’, ‘oncology’, or ‘malignancy’ to precisely identify relevant studies. Secondly, only independent research studies were considered, excluding meta‐analysis and reviews. Third, biomedical named entity recognition (NER) was performed on the extracted PubMed abstracts using PubTotor [41], identifying various biology terms, including miRNA, gene, cancer, and study species. Figure 1B provides a systematic overview of miR‐21 research across different cancers within all species and cell lines, conducted between January 1, 2010, and August 15, 2023. Our investigation unveiled a total of 781 studies related to miR‐21, highlighting the substantial interest this small RNA molecule has garnered in cancer research. Notably, miR‐21 has been most extensively studied in breast cancer, followed by colorectal cancer, lung cancer, hepatocellular cancer, gastric cancer, and others (Figure 1B). Finally, we focused exclusively on miR‐21 studies in breast cancer research involving Homo species for subsequent differential gene analysis and signaling pathway analysis.

### 3.2 Text Mining Identified miR‐21 Study in Breast Cancer

Text mining effectively identified a substantial number of studies related to miR‐21 in breast cancer, providing a rich dataset for analysis. From this dataset, we focused on a subset of 62 studies specifically exploring the role of miR‐21 in breast cancer, with particular emphasis on genes mentioned in the research abstract (Figure 1C). While the number of studies on miR‐21 in tumors was limited in 2010, it was not until 2018 that research on miR‐21 in cancer, particularly breast cancer, began to gain significant attention. The year 2020 marked a pivotal point, with a notable increase in both interest and research output on this topic (Figure 1C). Our findings reveal a substantial body of literature, with 55 publications focusing on human studies, underscoring the clinical significance of miR‐21 in breast cancer.

### 3.3 Identification of 5 Clusters of miR‐21 in Regulation of Breast Cancer in Human Studies

**FIGURE 2:**
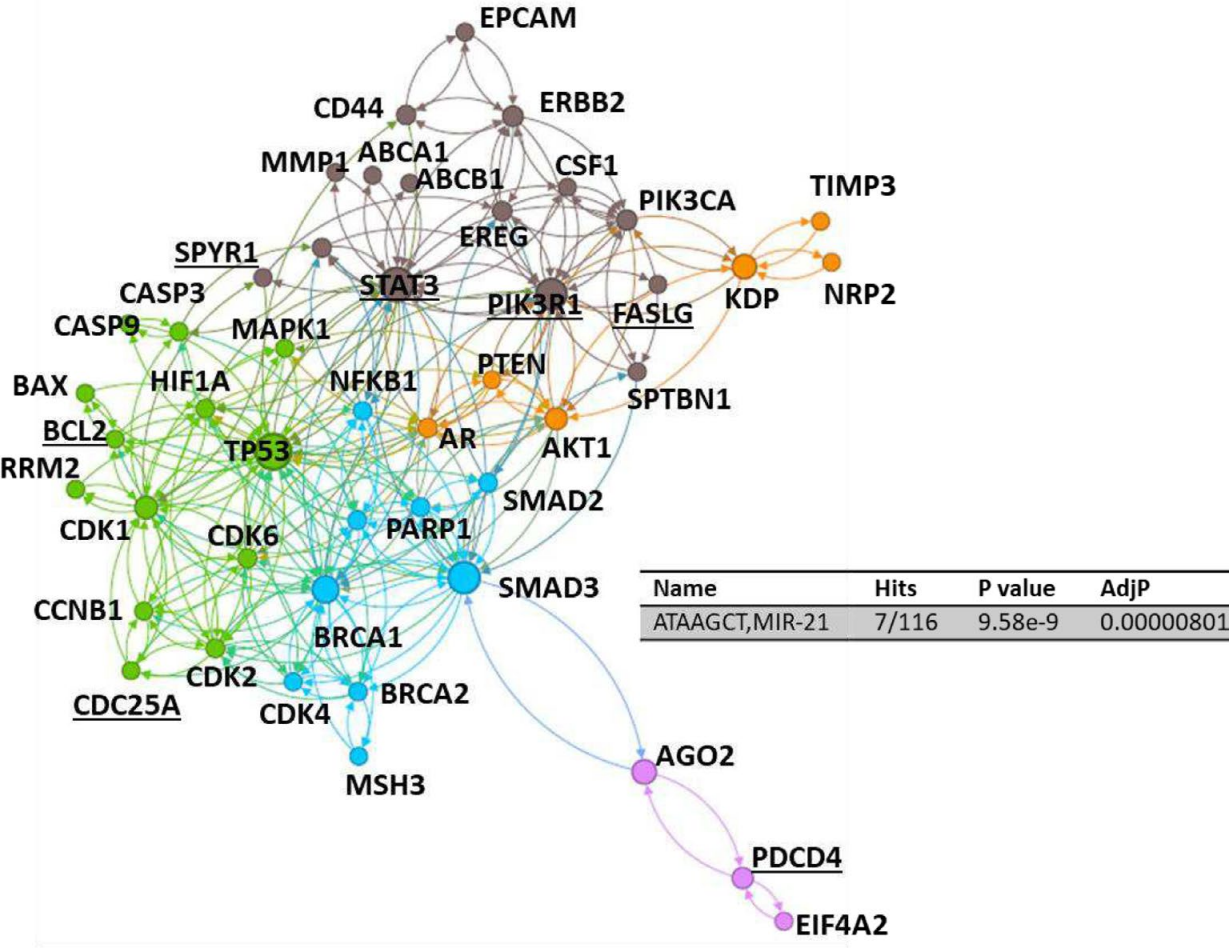
Genes identified from miR‐21 study in breast cancer research. The network illustrates five distinct clusters of genes, with seven genes marked as direct miR‐21 targets (underscore).

We employed PubTator, a powerful tool for extracting gene‐related terms from abstracts (Method). Our primary focus was on studies involving human subjects, ensuring the utmost relevance to breast cancer in the clinical context. Through this approach, we identified 79 unique genes (Table S1) that prominently featured in miR‐21‐related studies within the domain of human breast cancer research, many of which appeared in multiple studies: Phosphatase and tensin homolog (PTEN) [49, 50, 51, 52, 53, 54, 55, 56, 57, 58, 59, 60, 61, 62, 63], protein kinase B (AKT) [49, 50, 56, 60, 64, 65, 66], Human epidermal growth factor receptor 2 (HER2) [49, 67, 68, 69, 70, 71, 72], Estrogen receptor (ER) [62, 68, 73, 74, 75, 76], B‐cell lymphoma 2 (BCL2) [61, 62, 77, 78, 79], Programmed cell death protein 4 (PDCD4) [61, 62, 75, 80, 81], Progesterone receptor (PR) [68, 72, 74, 82], Transforming growth factor‐beta (TGF‐beta) [55, 83, 84, 85], Carcinoembryonic antigen (CEA) [82, 86, 87], Epidermal growth factor receptor 2 (EGFR2) [49, 74, 88], Cancer antigen 15‐3 (CA15‐3) [69, 86], Caspase 3 (CASP3) [60, 81], Vascular endothelial growth factor (VEGF) [51, 89], and BCL2‐associated X protein (BAX) [78, 79]. This recurrence indicated their critical roles in miR‐21's regulatory influence on breast cancer. Additional targets within this rich landscape, including Zinc finger protein 367 (ZNF367) [90], Fas ligand (FASLG) [91], Sprouty homolog 1 (SPRY1) [92], Signal transducer and activator of transcription 3 (STAT3) [93], Phosphoinositide‐3‐kinase regulatory subunit 1 (PIK3R1) [64], Cell division cycle 25A (CDC25A) [76], Ankyrin repeat domain‐containing protein 46 (ANKRD46) [94], Cyclin‐dependent kinase 6 (CDK6) [76], Mitogen‐activated protein kinase 1 (MAPK1) [91], and tumor protein p53 (TP53) [72], among others. To discern the direct targets of miR‐21, a motif analysis was performed using NetworkAnalysis(miRTarBase v9.0) [95], identifying a total of seven hits exhibiting supplementary binding seeds with miR‐21 (ATAAGCT), which includes PIK3R1, STAT3, CDC25A, FASLG, PDCD4, SPRY1, and BCL2 (Figure 2). To gain a comprehensive view of the gene networks regulated by miR‐21 in breast cancer, we began by inputting our gene list (Table S1) into the STRING database [96] to generate a molecular interaction network (Table S2). These networks were then clustered and visualized using Gephi software (Method). Arrows on the edges indicate directed interactions, indicating potential regulatory influences or causal relationships between molecules. The colors represent distinct clusters, which is specific designed to detect densely connected regions within the network using Louvain method, specifically, Louvain algorithm is a community detection method designed to identify densely connected clusters within large networks by optimizing modularity [97]. This clustering approach highlights groups of genes that may be functionally related or co‐regulated within the context of miR‐21's influence in breast cancer. Our analysis unveiled the presence of five distinct gene network clusters, each with its unique set of interacting genes (Figure 2): PIK3R1‐STAT3 network, TP53 network, BRCA1‐SMAD3‐CDK2 network, PDCD4 network, and AKT1‐PTEN network. Notably, these pathways are interconnected. We highlighted the direct targets of miR‐21 with underscore, illuminating pivotal nodes that play a central role in miR‐21‐mediated regulation. Taken together, this analysis provides a comprehensive overview of miR‐21's multifaceted role in breast cancer, shedding light on the intricate gene networks it influences.

### 3.4 Identifying Potential Gene Pathways That Are Regulated by miR‐21 in Breast Cancer

**FIGURE 3:**
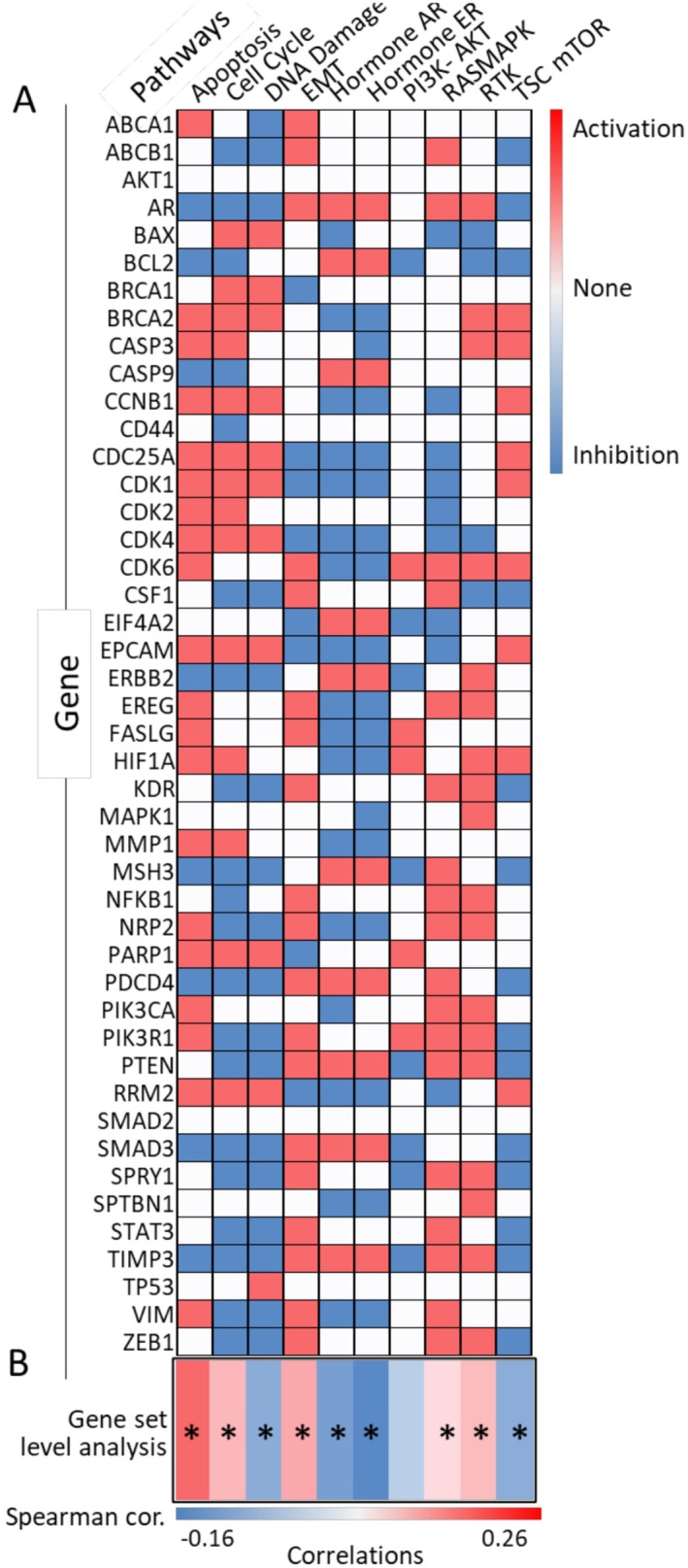
Gene pathways enrichment analysis with genes identified from miR‐21 study in breast cancer research. (A) Summarizes the gene presentation in corresponding pathways in breast cancer, red color represents the specific gene that plays an activation protentional effect in the pathway, the blue color represents the inhibition potential effect in the pathway, colors represent no protentional effect in this pathway. (B) Heatmap showing the association between GSVA (Gene Set Variation Analysis) score and activity of cancer‐related pathways in breast cancer. *p value < 0.05.

To gain a deeper understanding of the pathways orchestrated by miR‐21 in breast cancer, we conducted a gene set cancer analysis in breast cancer using GSCA(method) [47]. The heatmap visually represents the individual genes involved in significant pathways, with genes holding activation potential depicted in red and those with inhibitory potential in blue. Notably, our analysis revealed significant enrichment in pathways associated with Apoptosis (*p* value = 9.39781E‐16; cor = 0.26), Cell cycle (*p* value = 0.0000751; cor = 0.13), DNA Damage (*p* value = 0.008980131; cor = −0.09), Epithelial‐mesenchymal transition (EMT) signaling (*p* value = 2.08061E‐06; cor = 0.16), Hormone androgen receptor (AR) (*p* value = 0.008980131; cor = −0.10), Hormone estrogen receptor (ER) (*p* value = 0.000077657; cor = −0.13), RAS MARK (*p* value = 0.027641587; cor = 0.07), Receptor Tyrosine Kinase (RTK) (*p* value = 0.000182552; cor = 0.12), and the Tuberous Sclerosis Complex/mammalian Target of Rapamycin (TSC/mTOR) (*p* value = 0.010318309; cor = −0.09) (Figure 3). These enriched pathways align closely with the observed pathway changes in TCGA dataset [98], which encompasses the top 500 most DEGs between healthy tissue and breast cancer tissue (Table S3). This convergence of findings underscores the significance of miR‐21 in orchestrating crucial pathways implicated in breast cancer. Collectively, these pathway enrichment findings demonstrate the diverse molecular landscapes sculpted by miR‐21 in breast cancer.

### 3.5 Expression Patterns of miR‐21‐Related Genes in Healthy and Breast Cancer Tissue From TCGA

**FIGURE 4:**
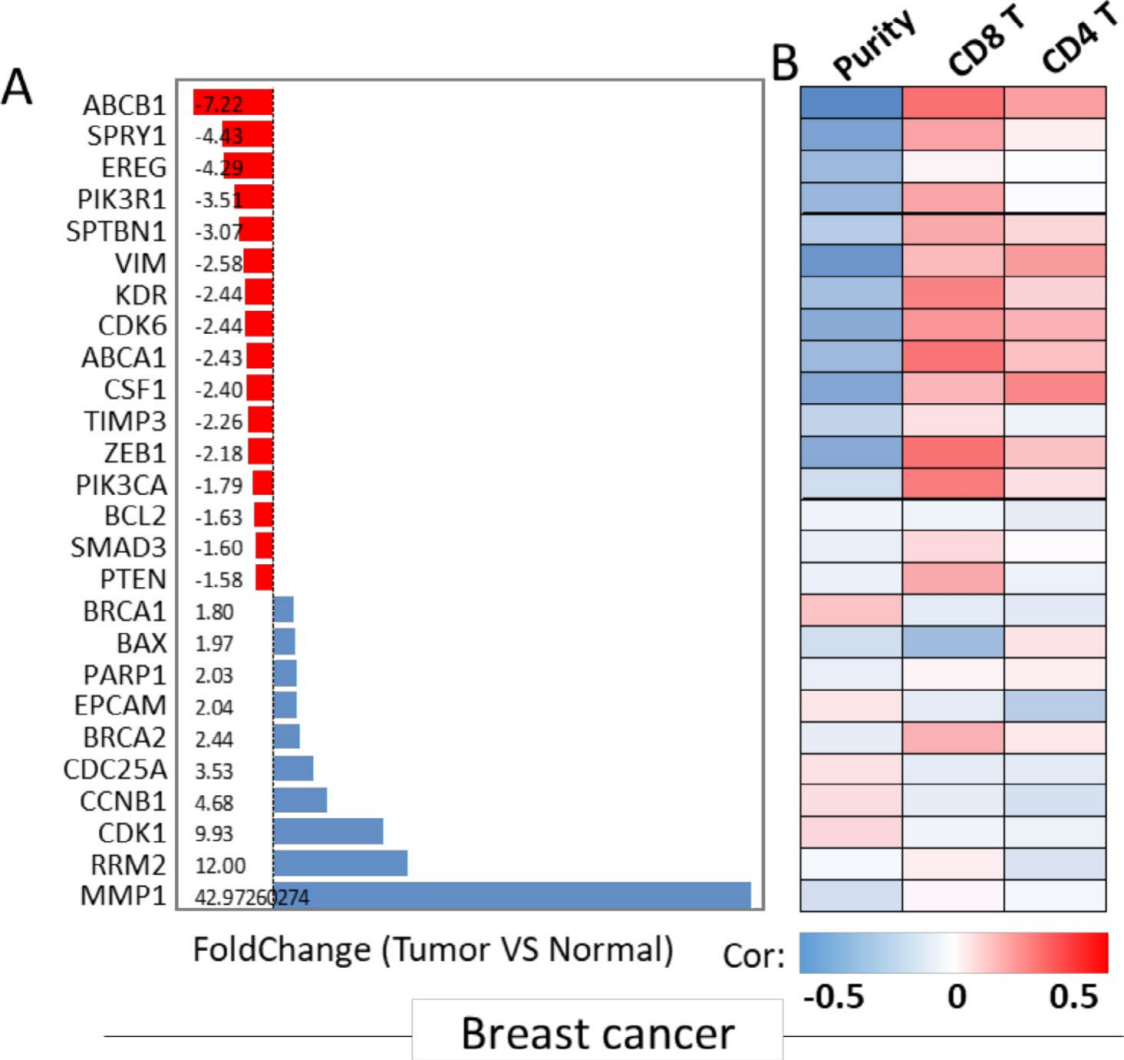
DEGs between breast cancer and normal from TCGA (A) The bar graph summarizes the DEGs with |FC| ≥ 1.5 between tumor and normal samples in breast cancer. (B) The heatmap shows the correlation between the corresponding genes with tumor purity, CD8+ T cell infiltration, and CD4+ T cell infiltration in the tumor microenvironment.

To gain a deeper understanding of the importance of miR‐21‐related genes in breast cancer development, we examined the expression patterns of these genes in normal and breast cancer tissues using the TCGA dataset. As depicted in Figure 4A, we observed significant changes in 26 out of all the genes featured in the pathway network, with |FC| > 1.5. Specifically, the genes ATP‐binding cassette sub‐family B member 1 (ABCB1), SPRY1, Epiregulin (EREG), PIK3R1, Spectrin beta chain, non‐erythrocytic 1(SPTBN1), Vimentin (VIM), Kinase insert domain receptor (KDR), Cyclin‐dependent kinase 6 (CDK6), ATP‐binding cassette sub‐family A member 1 (ABCA1), Colony stimulating factor 1 (CSF1), Tissue inhibitor of metalloproteinases 3 (TIMP3), Zinc finger E‐box‐binding homeobox 1 (ZEB1), Phosphatidylinositol‐4,5‐bisphosphate 3‐kinase catalytic subunit alpha (PIK3CA), BCL2, SMAD family member 3 (SMAD3), and PTEN exhibited significant downregulation. On the other hand, Breast cancer type 1 susceptibility protein (BRCA1), BAX, Poly [ADP‐ribose] polymerase 1 (PARP1), Epithelial cell adhesion molecule (EPCAM), Breast cancer type 2 susceptibility protein (BRCA2), CDC25A, Cyclin B1 (CCNB1), Cyclin‐dependent kinase 1 (CDK1), Ribonucleoside‐diphosphate reductase subunit M2 (RRM2), and Matrix metalloproteinase 1 (MMP1) were significantly upregulated in breast cancer tissue compared to normal tissue. These findings provide valuable insights into the altered expression patterns of miR‐21‐related genes in the context of breast cancer, emphasizing their potential roles in the disease's development.

### 3.6 The Relationship Between Mir‐21's Related Genes, Tumor Purity, and T‐Cell Infiltration

In the realm of cancer research, the integration of molecular data has become pivotal in unraveling the complexities of tumor biology. Utilizing the TIMER tool [99] has provided a comprehensive understanding of the intricate interplay between miR‐21 and a specific set of DEGs in breast cancer and normal samples. This curated gene list has undergone meticulous scrutiny to reveal its correlation with crucial aspects of tumor microenvironment dynamics, particularly tumor purity and T‐cell infiltration. As shown in Figure 4B, the top negative DEGs, including ABCB1, SPRY1, EREG, PIK3R1, SPTBN1, VIM, KDR, and others, exhibit a negative correlation with tumor purity while demonstrating a strong positive correlation with T‐cell infiltration, especially CD8+ T cells. Conversely, DEGs associated with an increase in tumor, such as RRM2, CDK1, and CCNB1, display a mild correlation with purity, with the majority showing a positive correlation with tumor purity but no correlation with T‐cell infiltration, including both CD8+ T cells and CD4+ T cells. The identified genes not only shed light on the potential role of miR‐21 in breast cancer pathogenesis but also contribute valuable insights into the intricate network of molecular players governing the tumor‐immune landscape.

## 4 Discussion

miR‐21 has been widely recognized as a potential therapeutic target in various cancers, including breast cancer, where its regulation of key genes significantly impacts cancer progression [10]. In this study, we employed new text mining and NLP techniques, focusing on recent publications from 2010 onwards to identify a comprehensive set of genes potentially regulated by miR‐21 in breast cancer. While individual studies may be limited by narrow focus, sample size, or study design, large‐scale text mining allows for the integration of findings across a wide range of publications. This approach helps identify consistently reported patterns, such as frequently studied miRNAs or common gene associations, which may not be apparent in isolated studies. By aggregating and analyzing data from numerous independent sources, text mining can reduce the impact of individual study bias and highlight broader trends in the literature. Importantly, this analysis allowed us to construct a detailed miR‐21‐gene network, offering valuable insights into the gene regulatory landscape associated with miR‐21 in breast cancer, with this, we identified 79 genes prominently featured in miR‐21 studies in human studies, with 9 genes were also found in mouse studies, highlighting the translational relevance of miR‐21 research (Table S1, highlighted with *). Pathway enrichment analysis revealed that miR‐21 impacts a diverse array of biological processes, including apoptosis, cell cycle regulation, and DNA damage response. Key signaling pathways affected by miR‐21 include EMT, androgen receptor (AR) signaling, estrogen receptor (ER) signaling, RAS/MAPK, RTK, and mTOR/TSC pathways, all of which are vital in breast cancer progression. Moreover, our analysis of miR‐21‐regulated genes has revealed intriguing correlations in breast tumor. Genes downregulated in tumor exhibited a negative correlation with tumor purity and a positive correlation with T cell infiltration, particularly with CD4+ T and CD8+ T cells. Conversely, genes upregulated in tumor showed a reverse correlation with tumor purity and T cell infiltration. These findings underscore the dynamic interplay between miR‐21 and the immune landscape of the tumor, offering valuable insights into miR‐21's potential immunomodulatory role in breast cancer progression.

Breast tumor growth is not solely a consequence of unbridled proliferation but also a result of diminished apoptosis. The dynamic interplay between these processes plays a pivotal role in dictating the overall trajectory of tumor growth or regression [100, 101, 102]. In this study, we found that miR‐21 is intricately involved in apoptosis and cell cycle regulation through its modulation of several genes, including but not limited to AR, BCL2, BRCA2, CASP3, CASP9, CCNB1, CDC25A, CSK1, Cyclin‐Dependent Kinase 2 (CDK2), CDK4, EPCAM, ERBB2, MMP1, MSH3, PARP1, etc. EMT, a fundamental process during embryonic morphogenesis, becomes co‐opted by tumor cells to execute the multifaceted stages of tumorigenesis and metastasis. Numerous transcription factors and signaling cascades orchestrate these events [103, 104, 105]. In our investigation, we observed that miR‐21's regulatory influence on pivotal genes involved in EMT, such as ABCA1, ABCB1, AR, CSK6, CSF1, EREG, FASLG, KDR, Nuclear Factor Kappa B Subunit 1(NFKB1), NRP2, PDCD4, PIK3R1, PTEN, SMAD3, SPRY1, STAT3, TIMP3, VIM, and ZEB1. These genes are central to signaling pathways that govern cell polarity, adhesion, migration, and immune evasion [106, 107, 108, 109, 110]. Furthermore, miR‐21 demonstrated involvement in hormone receptor pathways, especially the AR and ER pathways, the co‐expression of which significantly contributes to the development of ER‐positive breast cancer [111, 112]. The aberrant activity of the Ras/MAPK pathway has been identified as pivotal in the initiation and progression of breast cancer [113], miR‐21's targeting of key activators within this pathway, including ABCB1, AR, CDK6, CSF1, EREG, MKDR, among others. Additionally, miR‐21‐related genes augment the RTK pathway while inhibiting the TSC/mTOR pathway. RTKs, well‐known for orchestrating downstream signaling cascades, such as MAPK, PI3K/Akt, and JAK/STAT, have been substantiated in a separate study [92]. These pathways have a pivotal role in the regulation of cancer stemness, particularly in the context of breast cancer [114, 115]. The frequently observed dysregulation of the TSC/mTOR pathway in breast cancers further underscores its association with tumorigenesis [116, 117]. Notably, several validated targets of miR‐21, such as PTEN, are key negative regulators of the PI3K/AKT/mTOR signaling axis. Downregulation of these targets by miR‐21 can result in hyperactivation of the mTOR pathway, promoting tumor growth and survival. Given this mechanistic link, targeting miR‐21 may restore the expression of these negative regulators, thereby sensitizing tumors to mTOR inhibitors. Indeed, previous studies have suggested that combining miR‐21 inhibition with mTOR pathway blockade could produce synergistic antitumor effects [118].

T cells, particularly CD8+ T cells, play a central role in recognizing and eradicating cancerous cells. As they infiltrate the tumor microenvironment, CD8+ T cells have the capability to directly target and eliminate malignant cells by releasing cytotoxic molecules [119]. This immune response is pivotal for effectively controlling tumor growth and thwarting the onset of metastasis [119]. Our study discerned that miR‐21‐related genes, such as ABCB1, SPRY1, ERGE, PIK3R1, SPTBN1, VIM, and others, which experienced downregulation in tumor sites, exhibited a negative correlation with tumor purity and a positive association with T cell infiltration, notably CD8+ T cells. This pattern suggests that these genes may be predominantly expressed by immune or stromal cells within the tumor microenvironment, rather than by tumor cells themselves. The inverse relationship with tumor purity implies that as the proportion of malignant cells increases, the expression of these genes decreases—likely reflecting reduced immune cell presence. These findings support the notion that miR‐21 may play a role in modulating the immune landscape of breast cancer, potentially by regulating pathways involved in immune cell recruitment or activation, especially of cytotoxic CD8+ T cells. With that, experimental follow‐up studies are essential to validate the regulatory interactions predicted in our analysis. For instance, knockdown or overexpression of key miR‐21 target genes—such as SPRY1—in breast cancer cell lines or patient‐derived organoids could help confirm their functional roles in tumor progression and clarify the mechanistic impact of miR‐21 in breast cancer.

While miR‐21 is among the most frequently studied miRNAs in breast cancer, other miRNAs such as miR‐155, miR‐182, and the let‐7 family also play significant roles in breast cancer pathogenesis [27, 32, 120, 121, 122, 123, 124]. Functionally, miR‐21 shares overlapping oncogenic roles with miR‐155 and miR‐182, particularly in promoting cell proliferation, survival, and immune modulation. All three miRNAs target tumor suppressor genes and are frequently upregulated in aggressive breast cancer subtypes. However, their regulatory networks are partially distinct—miR‐155 is more closely linked with inflammatory signaling and immune cell differentiation [32, 125], while miR‐182 is heavily implicated in DNA repair suppression and metastasis [123, 126]. In contrast, let‐7 acts as a tumor suppressor and may counterbalance the effects of miR‐21 by inhibiting oncogenes such as RAS and MYC [124]. These dynamics suggest possible synergistic interactions between miR‐21, miR‐155, and miR‐182 in driving tumor progression, while antagonistic interactions may exist between miR‐21 and let‐7, especially in the regulation of cell cycle and apoptosis pathways. Additionally, miR‐21 itself plays a dual role in tumor formation and cytotoxic response in breast cancer [127]. Exploring these combinatorial effects could provide deeper insight into miRNA network dynamics and help identify more effective multi‐target therapeutic strategies in breast cancer.

Our study is an innovation in two areas: (1) utilizing text mining has facilitated the extraction of bio terms, including genes and species from miR‐21 studies in breast cancer; and (2) with this methodology, we identified the comprehensive networks of the miR‐21‐gene‐signaling pathway based on 2010–2023 studies on miR‐21 in breast cancer. This empowers researchers to draw robust and broadly applicable conclusions, contributing to a more comprehensive understanding of the intricate network between miR‐21 and breast cancer based on current literature data. Nonetheless, it is important to acknowledge the limitations of our current study: Our approach, which covers studies from 2010 to 2023, focused on breast cancer and extracted data from abstracts containing gene names and species information. Our decision to focus on papers from 2010 onwards was made to prioritize more recent findings and technological advancements, which may offer a better reflection of current understanding and trends in miRNA research. We recognize that valuable earlier studies were published as early as 2005 [128], while this study is more focused on miRNA expression profiles in breast cancer but lacked detailed gene target regulation. Furthermore, as it is primarily based on text mining from published studies, further deep analysis for clinic therapy translation is needed. Firstly, improvements in text mining methodologies are necessary, particularly in screening full‐text manuscripts and refining sentence structure analysis to identify more comprehensive miRNA‐gene interactions in cancer. Secondly, the interaction between miR‐21 and other regulatory elements, such as long non‐coding RNAs and transcription factors, remains a promising area for future exploration. Elucidating these complex regulatory networks could provide a more comprehensive understanding of breast cancer pathogenesis. Lastly, the clinical utility of miR‐21 as a clinical therapy target in breast cancer should focus more on the treatment mode and curative effect, which requires the network identified in our study to be further validated in experimental studies. For instance, previous studies have demonstrated that miR‐21 regulates PDCD4 expression in breast cancer [61, 62, 75, 80, 81], and PDCD4 is considered a prognostic marker for breast cancer [129]. Additionally, miR‐21 has been implicated in regulating apoptosis pathways [81] and resistance to paclitaxel by targeting on PDCD4 [80]. Our integrative analysis suggests that PDCD4 may also be involved in the EMT pathway during breast cancer development, a hypothesis that warrants further validation through experimental studies.

It is important to acknowledge that earlier study [130] has made significant contributions to miRNA research using text mining, particularly in identifying miRNA‐cancer associations across a range of cancers. These foundational efforts have provided a broad understanding of miRNA profile in cancer, which however overlooked the specific miRNA‐target gene interactions within individual cancers.

In conclusion, this comprehensive analysis of miR‐21's role in breast cancer reveals its extensive regulatory networks and enriched pathways, not only deepen our understanding of breast cancer pathogenesis but also pave the way for potential therapeutic strategies.

Future work should focus on experimentally validating miR‐21‐associated pathways, particularly its link to CD8+T cell infiltration and immune regulation. Combining text‐mined insights with patient‐derived multi‐omics data may enhance clinical relevance and support the development of miRNA‐based therapies and personalized approaches in breast cancer.

## Author Contributions

**Hong Ye:** data curation (lead), formal analysis (lead), visualization (equal), writing – original draft (lead). **Yuyu Wu:** formal analysis (equal), investigation (equal), methodology (equal). **Richard Tran:** funding acquisition (equal), investigation (equal), methodology (equal). **Jie Wang:** methodology (equal), resources (equal), writing – original draft (equal), writing – review and editing (equal).

## Ethics Statement

The authors have nothing to report.

## Conflicts of Interest

The authors declare no conflicts of interest.

## Supporting information


Table S1.

Table S2.

Table S3.

## Data Availability

Researchers interested in accessing the data for research purposes can contact Jie Wang (jwang326@syr.edu). The statistical codes used in this work will be made accessible upon reasonable request to the author.
